# Development of melanopsin-based irradiance detecting circuitry

**DOI:** 10.1186/1749-8104-6-8

**Published:** 2011-03-18

**Authors:** David S McNeill, Catherine J Sheely, Jennifer L Ecker, Tudor C Badea,, Duncan Morhardt, William Guido, Samer Hattar

**Affiliations:** 1 Department of Biology, Johns Hopkins University, Baltimore, MD 21218, USA; 2 Department of Anatomy and Neurobiology, Virginia Commonwealth University, Richmond, VA 23298, USA; 3 Retinal Circuit Development and Genetics Unit, N-NRL/NEI/NIH, Bethesda, MD 20892-0610, USA

## Abstract

**Background:**

Most retinal ganglion cells (RGCs) convey contrast and motion information to visual brain centers. Approximately 2% of RGCs are intrinsically photosensitive (ipRGCs), express melanopsin and are necessary for light to modulate specific physiological processes in mice. The ipRGCs directly target the suprachiasmatic nucleus (SCN) to photoentrain circadian rhythms, and the olivary pretectal nucleus (OPN) to mediate the pupillary light response. How and when this ipRGC circuitry develops is unknown.

**Results:**

Here, we show that some ipRGCs follow a delayed developmental time course relative to other image-forming RGCs. Specifically, ipRGC neurogenesis extends beyond that of other RGCs, and ipRGCs begin innervating the SCN at postnatal ages, unlike most RGCs, which innervate their image-forming targets embryonically. Moreover, the appearance of ipRGC axons in the OPN coincides precisely with the onset of the pupillary light response.

**Conclusions:**

Some ipRGCs differ not only functionally but also developmentally from RGCs that mediate pattern-forming vision.

## Background

The mammalian retina detects, processes, and signals light information to the brain to form images and modulate physiological processes. As the sole output neurons of the retina, most retinal ganglion cells (RGCs) encode visual information such as motion and contrast, whereas 2% of RGCs contain the photopigment melanopsin and signal irradiance information to the brain to control circadian rhythms, sleep, and the pupillary light response (PLR) [1-4]. These intrinsically photosensitive RGCs (ipRGCs) project directly to brain regions that mediate light-dependent physiological processes, notably the suprachiasmatic nucleus (SCN), which is the master circadian pacemaker, and the olivary pretectal nucleus (OPN), which controls PLR [5,6]. Genetic ablation of ipRGCs leaves image formation intact, but severely impairs the effects of light on circadian rhythms, sleep, and PLR [3,4]. Thus, the pathways for light input to image-formation and regulation of physiological processes diverge at the level of the RGCs.

Until recently, the majority of research on ipRGCs has focused on the initially identified subtype (M1), which arborizes in the OFF sublamina of the inner plexiform layer and labels with expression of the tauLacZ reporter from the endogenous melanopsin locus (*Opn4^taulacZ^*). However, a more sensitive reporter system utilizing expression of cre recombinase from the endogenous melanopsin locus (*Opn4^cre^*) revealed additional subtypes of ipRGCs beyond the previously identified M1 subtype. Some of these newly identified subtypes target the dorsal lateral geniculate nucleus (dLGN), a thalamic relay center for visual processing. This work also demonstrated that ipRGCs are capable of supporting visual processing, indicating that some ipRGCs have overlapping targeting and function with other RGCs [7].

How ipRGCs develop in order to fulfill their unique physiological functions remains unclear. To begin answering this question, we examined the timing of ipRGC neurogenesis, axonal targeting, and the developmental onset of pupil constriction, an ipRGC-mediated functional output.

## Results

### Birth of ipRGCs continues beyond that of other RGCs

**Figure 1:**
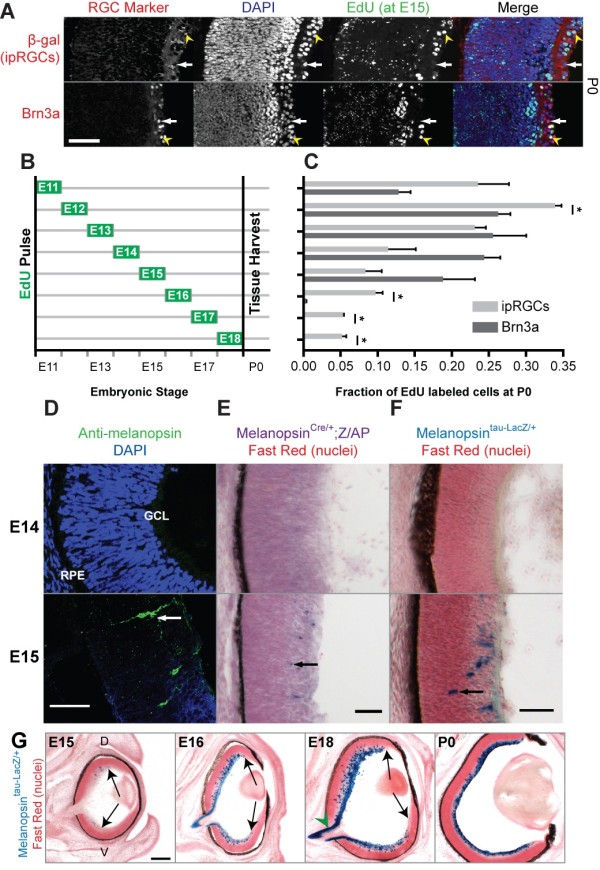
**Birth and melanopsin expression of ipRGCs**. **(A,B) **The birthdates of ipRGCs were compared to Brn3a-positive RGCs (representative images in (A), paradigm in (B)). **(C) **For each time point, we determined the proportion of ipRGCs labeled with EdU; asterisks indicate significant difference between ipRGCs and Brn3a (*t*-test, *P *< 0.05). Representative sections with double-labeled RGCs (yellow arrowheads) and EdU-negative RGCs (white arrows) from postnatal day 0 (P0) retinas pulsed with EdU at embryonic day 15 (E15) are shown in (A). Note that the ipRGC marker beta-galactosidase (β-gal), is cytoplasmic, while Brn3a and EdU are nuclear. For all EdU time points, n = 3 to 4 retinas per time point, mean ± standard error of the mean. **(D-F) **Melanopsin (*Opn4*) expression begins at E15 based on immunofluorescence (D), and the Melanopsin^*Cre/+*^;*Z/AP *(E) and *Melanopsin^tauLacZ/+ ^*genetic labeling methods (F). The Melanopsin^*Cre/+*^;*Z/AP *labels all ipRGC subtypes and does not depend on the melanopsin locus for signal strength. Arrows in D-F denote migrating cells. GCL; Ganglion Cell Layer and RPE; Retinal Pigmented Epithelium. **(G) **Coronal sections from *Melanopsin^tauLacZ/+ ^*mice show an initial lack of ipRGCs in the periphery at E15, which is entirely filled in by P0 (arrows; D, dorsal; V, ventral). Note the lack of X-gal staining within the central optic nerve at E18 (G, green arrowhead). Scale bars: 50 μm (A,D-F); 100 μm (G).

To compare ipRGC neurogenesis with other RGCs, we labeled terminally dividing cells with 5-ethynyl-2'-deoxyuridine (EdU) on individual days of embryonic development (Figure 1A,B). We used immunostaining for the transcription factor Brn3a to label a subset of RGCs separate from ipRGCs [8] and the melanopsin-tau-LacZ (*Opn4^tauLacZ/+^*) reporter to detect ipRGCs. The tau-lacZ reporter expresses tau-β-galactosidase, which is cytoplasmic and is predominantly trafficked to axons. The *Opn4^tauLacZ/+ ^*reporter primarily labels the M1 subtype in adult mice [9], but it remains unclear if this holds true during development since ipRGC subtypes cannot be anatomically identified prior to dendritic arborization. Tissue was analyzed at postnatal day 0 (P0), which is a time point after RGC birth but before the major wave of RGC death [10,11]. The onset of ipRGC neurogenesis is consistent with that observed for Brn3a-positive RGCs as well as the RGC population as a whole, which begins at embryonic day 11 (E11) [11,12]. EdU injections on subsequent days during development revealed that the majority of ipRGCs are born from E11 to E14 (Figure 1C; Additional file 1). Although the Brn3a population shows a sharp decline in birth after E15, with only one positive cell observed at E16, a significant proportion of ipRGCs are born through E18 (Figure 1B,C).

To determine when melanopsin is first expressed in the developing retina, we used three different labeling methods: a melanopsin antibody and two genetic reporter mouse lines, *Opn4^tauLacZ/+ ^*[5] and *Opn4^Cre/+ ^*in conjunction with the *Z/AP *reporter, which does not depend on transcription of the melanopsin locus for signal strength [7,13]. All three labeling techniques indicated that melanopsin is expressed from E15 onwards since there was a complete absence of staining at E14 (Figure 1D-F). Melanopsin expression is first detected at a similar time point in rats [14]. While it is possible that melanopsin is expressed at low levels prior to E15, it is unlikely because the genetic reporters used in this study have high sensitivity due to enzymatic signal amplification. At E15 some melanopsin-positive cells were detected in the ganglion cell layer, while others appeared to be migrating from the neuroblast layer to the ganglion cell layer (Figure 1D-F, arrows). Although melanopsin positive cells were absent from the peripheral retina at E15, during subsequent days of development the region of labeled cells subsequently expanded (Figure 1G, arrows), reaching the ciliary margin by P0.

### ipRGCs innervate their main target, the suprachiasmatic nucleus, postnatally

**Figure 2:**
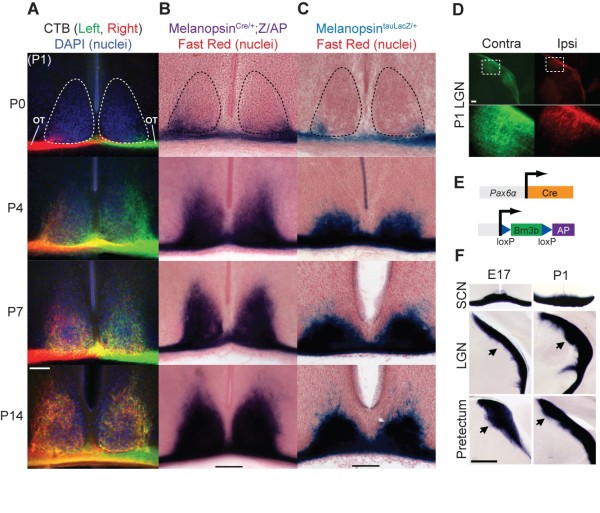
**ipRGCs innervate the suprachiasmatic nucleus postnatally**. Innervation of the suprachiasmatic nucleus (SCN) begins at P0 and continues throughout the first two postnatal weeks. **(A) **Labeling of all RGCs in coronal sections with fluorescently labeled cholera toxin B subunit (CTB), one color per eye. Retinal fibers fill the SCN by P7 and innervation becomes bilateral by P14. Note that for CTB labeling, a P0 time point was not possible due to the survival period required for the tracer to label distal axons. **(B,C) **Labeling of ipRGCs and their axons with both Melanopsin^*Cre/+*^;*Z/AP *(B) and *Melanopsin^tauLacZ/+ ^*(C) reveals a similar innervation pattern. **(D-F) **In contrast, CTB labeling reveals RGC axons have already penetrated the LGN by P1 (D), and genetic labeling of Brn3b-positive RGCs (E) reveals that this subset of RGCs begins entering the pretectum as early as E17 (F). Arrows indicate fibers entering target area. n = 3 or more animals for each time point. OT, optic tract; dashed outlines demarcate the SCN prior to innervation in (A,B). Scale bars: 100 μm (A-C); 500 μm (F).

**Figure 3:**
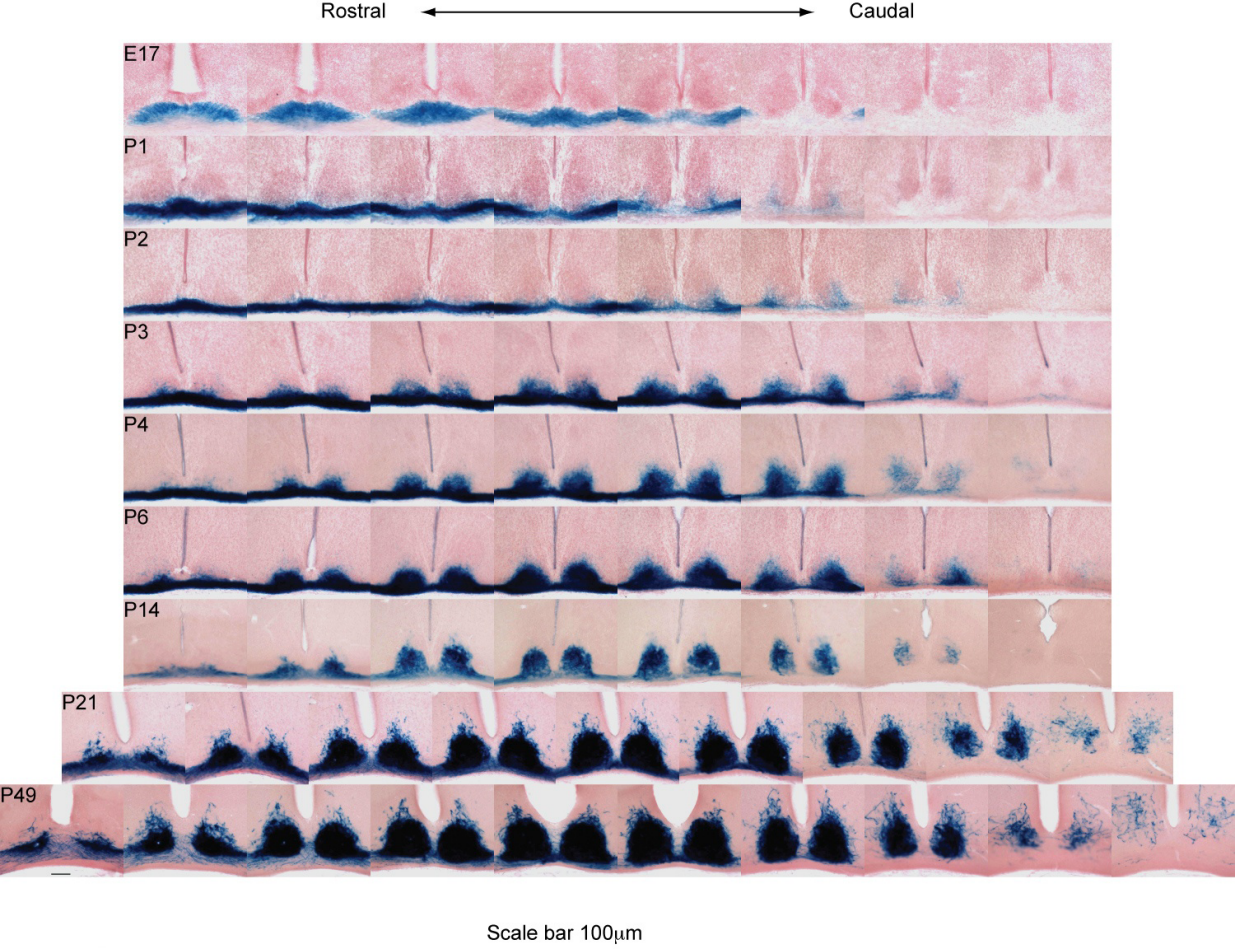
**Spatial and temporal progression of SCN innervation**. Serial 50-μm coronal sections from individual brains of *Melanopsin^tauLacZ/+ ^*mice at different time points reveal the spatial and temporal progression of ipRGC innervation of the SCN. Nuclei are counterstained with Vector Nuclear Fast Red. Scale bar: 100 μm.

**Figure 4:**
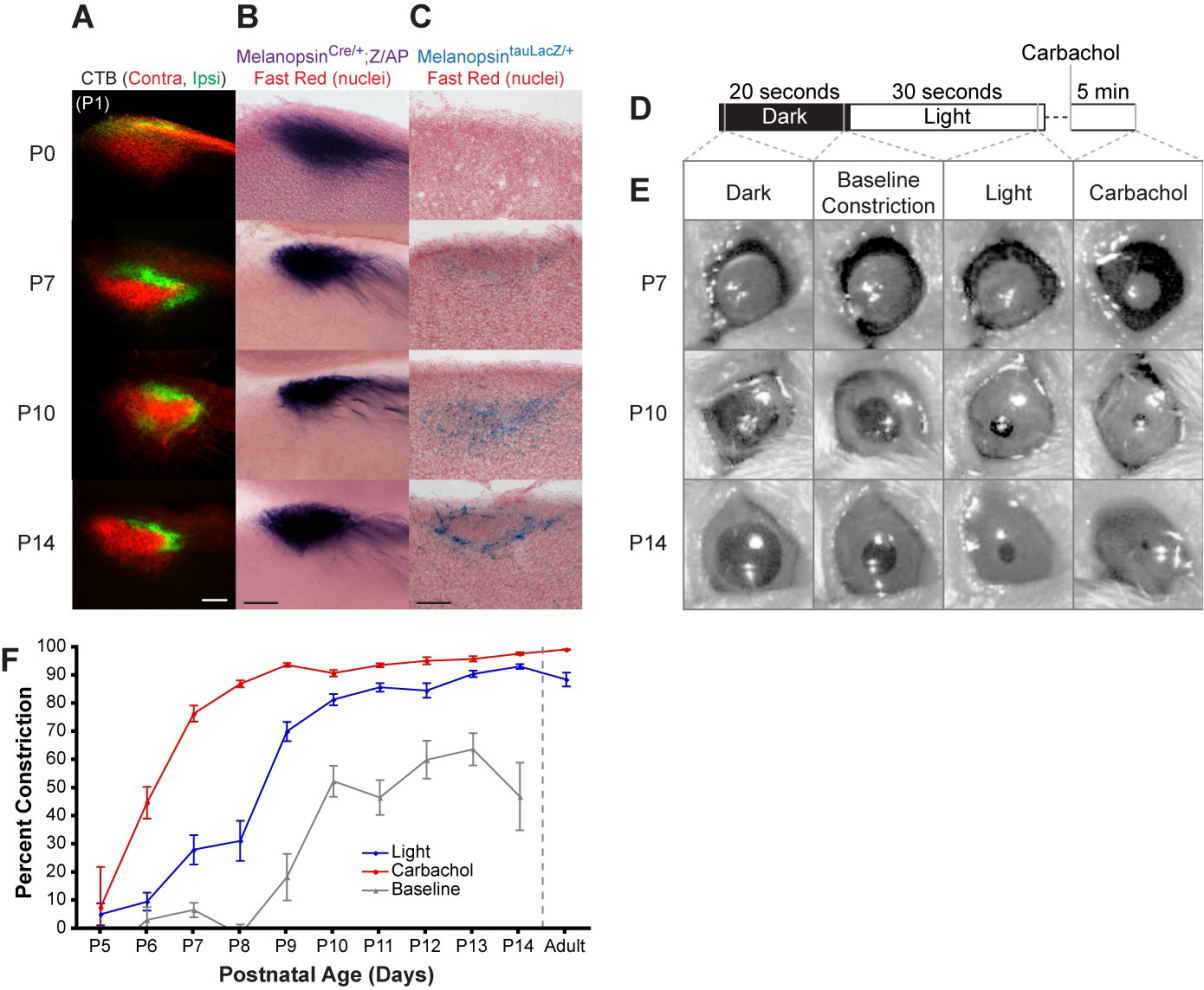
**Olivary pretectal nucleus innervation and onset of pupillary light responses**. **(A) **The olivary pretectal nucleus (OPN) is innervated by CTB-labeled RGC axons at P0 and ipsilateral and contralateral axons have segregated by P7. **(B) **Melanopsin^*Cre/+*^;*Z/AP*-labeled axons show a similar innervation pattern from birth. **(C) **In contrast, *Melanopsin^tauLacZ/+^*-labeled axons are not detected until P7 and show adult-like innervation of the OPN shell by P14. **(D,E) **The paradigm used for all pupil measurements (D) and representative images (E). Note that the pupil appears white at P7 because at this early time point it was necessary to reflect infrared light off the back of the eye to make the iris visible to the camera. Carbachol was used to induce maximal constriction of the ciliary muscle. **(F) **Percent constriction of pupil was measured and the summary of data for PLR is shown. Significant light responses are detected from P7 onward (n = 3 or more mice per time point, one-way ANOVA with Tukey post hoc test, error bars are mean ± standard error of the mean). No baseline constriction was observed in adult mice. Scale bars: 100 μm (A-C).

To determine when RGC axonal projections from the eye reach the SCN, we used four different labeling methods. Total RGC axonal projections were labeled with cholera toxin B subunit (CTB), which revealed that RGC axons begin to innervate the contralateral borders of the SCN soon after birth (Figure 2A), similar to rats [15,16]. At P3 to P4 axons from both eyes emerged at the midline to provide bilateral innervation of the SCN beginning caudally and achieving an adult-like pattern by the second postnatal week. To specifically label ipRGC axons, we used the *Opn4^tauLacZ/+ ^*mice, which directly report transcription from the melanopsin locus and the *Opn4^Cre/+^*;*Z/AP *reporter, which does not depend on the melanopsin locus for signal strength. The innervation patterns revealed by all three labeling methods are identical (Figure 2A-C) and directly correspond to the spatial and temporal progression of cFos induction in the SCN by light in early postnatal mice [17]. Even though the SCN is innervated postnatally (Figure 2A-C), ipRGC axons are present in the chiasm on the ventral surface of the SCN as early as E17 (Figure 3). In contrast, axons from cholera toxin labeled RGCs have already entered more caudal targets, such as the lateral geniculate nucleus and the pretectum by P1 (Figures 2D and 4A) [18]. To directly compare the innervation patterns of ipRGCs to a larger population of RGCs, we used an alkaline phosphatase reporter that labels RGCs positive for the transcription factor Brn3b (Figure 2E) [18]. We confirmed that the SCN is innervated later than visual targets such as the LGN, where fibers enter by P1, and the pretectum, where fibers enter by E17 (Figure 2F, arrows). Serial sections at different developmental time points reveal the complete spatial and temporal progression of *Opn4^tauLacZ/+^*-labeled axons in the SCN (Figure 3).

### Onset of the pupillary light response corresponds to emergence of ipRGC axons in the OPN shell

**Figure 5:**
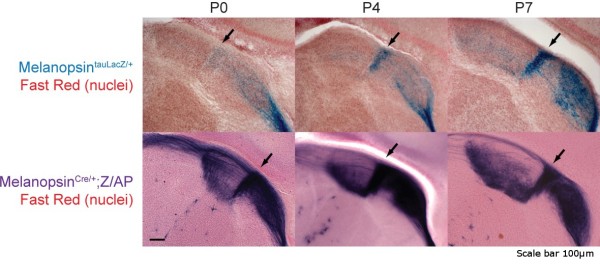
**ipRGC innervation of the LGN**. Comparison of LGN innervation by *Opn4^tauLacZ/+^*-labeled ipRGC axons and *Opn4^Cre/+^*;*Z/AP *labeling of total ipRGC axons across postnatal development. Arrows denote the intergeniculate leaflet, which separates the dorsal and ventral LGN. Scale bar: 100 μm.

Next we used CTB, *Opn4^tauLacZ/+^*, and *Opn4^Cre/+^*;*Z/AP *labeling techniques to determine the development of retinal projections to the OPN, which mediates the PLR [19]. Both CTB labeling of RGC axons and *Opn4^Cre/+^*;*Z/AP *labeling of ipRGC axons revealed innervation of the OPN from birth, achieving an adult-like morphology by P7 (Figure 4A,B). In contrast to this early postnatal labeling throughout the OPN (Figure 4A,B), the *Opn4^tauLacZ/+^*-labeled ipRGC axons showed only faint labeling in the shell of the OPN starting at P7 and reached adult morphology at P14 (Figure 4C). Similarly, *Opn4^Cre/+^*;*Z/AP*-labeled ipRGC axons are present throughout the LGN from birth, including the dorsal LGN, which is an important relay for visual information. In contrast, the *Opn4^tauLacZ/+^*-labeled ipRGC axons do not begin to appear in the intergeniculate leaflet of the LGN until about P3 (Figure 5). Thus, for both the OPN and the LGN, the *Opn4^tauLacZ/+^*-labeled ipRGC axons appear to innervate these regions later than the other ipRGC axons that label with the *Opn4^Cre/+^*;*Z/AP *reporter.

Next we determined if the appearance of ipRGC axons in the OPN correlates with the onset of the functional output of the OPN, the PLR. Under high light intensity (1.35 × 10^16 ^photons/cm^2 ^s), we observed rudimentary PLR starting at P7 and substantial responses from P10 onwards (representative images shown in Figure 4E, data summarized in Figure 4F). This onset of the PLR is similar to that in rats [20]. The lack of PLR prior to P7 was not limited by development of the ciliary muscle since from P6 to P8 the cholinergic agonist carbachol constricted the pupil significantly more than light (one-way ANOVA, *P *< 0.0001; Tukey post hoc, *P *< 0.001). From P10 onwards, the light intensity we used constricted the pupil to levels similar to carbachol, indicating that it is bright enough to cause maximal constriction (one-way ANOVA, Tukey post hoc, *P *> 0.05 for P10 to P14). Interestingly, we noticed a baseline constriction of the pupil in the dark before light exposure from P9 to P14, perhaps caused by holding the lid margins open (see Materials and methods and paradigm in Figure 4D). This constriction was always significantly less than the constriction due to light (one-way ANOVA, *P *< 0.0001; Tukey post hoc, *P *< 0.01 for P7 and *P *< 0.001 for P8 to P14). Application of the topical anesthetic proparacaine did not have a significant effect on this baseline constriction; thus, it was unlikely to be caused by the direct sensation of the forceps (data not shown). In conclusion, light consistently induced pupil constriction starting at P7, coincident with the appearance of ipRGC axons in the shell of the OPN, a crucial connection in the PLR circuit [4,9].

## Discussion

Over a century of research has focused on the development of RGCs and their role in image formation. Work over the last decade has revealed an intrinsically photosensitive subset of RGCs (ipRGCs) that signal irradiance information to brain regions that modulate processes such as sleep, circadian rhythms, and PLR [3,4]. Here, we provide the first direct examination of ipRGC birth and axonal targeting in the brain. Our data reveal a broad diversity of developmental parameters within the ipRGC population, with the development of some ipRGCs diverging from the majority of other RGCs.

### A subset of ipRGCs is born later than other RGCs

Previous studies have shown that RGCs are born between E11 and E18 [12,21]. We find that most ipRGCs are born between E11 and E15, similar to other RGCs [11,12,21]. In stark contrast to the Brn3a-positive RGCs, we observe a significant amount of ipRGC neurogenesis after E15 (Figure 1C). Interestingly, Math5, a transcription factor involved in RGC fate determination, is downregulated at E16 [22]. Thus, this late born ipRGC cohort may be specified independent of Math5. In agreement with this idea, some ipRGCs remain in the Math5 knockout [23]. The early born ipRGCs may share an overlapping function with other RGCs, such as the ipRGCs that are capable of supporting pattern vision [7], and the later born ipRGCs may preferentially target non-image forming areas that are innervated later, such as the SCN and the shell of the OPN. Such a divergence could be determined by the identification of molecular markers for individual ipRGC subtypes.

### The SCN is innervated later than image-forming retinal targets

During embryonic development, the majority of RGC axons project through the optic chiasm past the overlying SCN to target visual brain centers such as the LGN and the superior colliculus (Figure 2F) [15,16,24,25]. In contrast, cholera toxin labeling of RGC fibers showed that retinal axons enter the SCN postnatally. The lateral edges of the SCN fill first according to CTB labeling and both the *Opn4^tauLacZ/+ ^*and *Opn4^Cre/+^*;*Z/AP *reporters, which in turn closely match a previous report that exposure to bright light can induce cFos expression in the lateral edges of the SCN in newborn mice [17]. The spatio-temporal agreement of ipRGC axonal labeling with the induction of cFos expression by light supports the well-established idea that most of the RGCs that innervate the SCN express melanopsin [4-6,9,26].

There are at least three possible explanations for this later innervation of the SCN by ipRGCs. First, ipRGC axons may follow a later time course compared to other RGCs. Such a delay in targeting would correspond with the later born ipRGCs we observe. Second, ipRGC axons that innervate the SCN may reach the chiasm earlier in development, but stall there until P0. Though we do observe ipRGC axons in the chiasm ventral to the SCN from E17, it remains to be determined if these specific ipRGC axons subsequently innervate the SCN (Figure 3). It is doubtful that innervation of the SCN is dependent upon functional maturation of target cells since the SCN begins oscillating before birth [27,28]. Finally, the SCN could be innervated by collaterals from RGC axons that have already passed through the chiasm to more distal targets in the LGN, pretectum or colliculus [29,30]. Indeed, such a delay in collateralization of RGC axons into the SCN has previously been reported [15]. Since most RGC axons pass by the SCN to enter visual targets embryonically (Figure 2F), this temporal separation could be a means for preventing non-ipRGC axons from aberrantly terminating in the SCN. If this is the case, the ipRGCs that innervate the SCN may be uniquely receptive to a yet undetermined signal from the SCN. Expression of such factors may complement the spatial and temporal characteristics of ipRGC innervation of the SCN during development (Figure 3).

### Onset of PLR correlates with emergence of ipRGC axons in the OPN shell

The PLR is mediated by retinal input to the OPN, which can be divided into core and shell regions. The shell is defined by parvalbumin and calbindin-D expression [31,32], and is innervated by the M1 subset of ipRGCs, which label with *Opn4^tauLacZ/+ ^*[6,9]. This connection of M1 ipRGCs to the OPN shell is crucial to the PLR circuit, since genetic ablation of the *Opn4^tauLacZ/+^*-labeled ipRGCs results in severe impairment of the pupillary light response [4]. In agreement with these data, the Edinger-Westphal nucleus, which is the brain relay downstream of the OPN in the PLR circuit, is primarily innervated by axons from neurons in the shell of the OPN [9].

Our data suggest that two subtypes of ipRGCs innervate the OPN with different spatial and temporal profiles. CTB labeling of RGC axons and *Opn4^Cre/+^*;*Z/AP *labeling of all ipRGCs reveal robust innervation of the OPN from birth. In contrast, the *Opn4^tauLacZ/+ ^*reporter only shows labeling of ipRGC axons in the OPN starting in the second postnatal week. This delayed labeling is limited to the shell region of the OPN, similar to adult labeling with the *Opn4^tauLacZ/+ ^*reporter [5,6]. Thus, it appears that the M1 ipRGCs innervate the OPN shell later than the ipRGCs that innervate the core. We observe a similar temporal separation between *Opn4^Cre/+^*;*Z/AP*-labeled ipRGCs that innervate the LGN and the *Opn4^tauLacZ/+^*-labeled subset that innervate the intergeniculate leaflet (Figure 5). Thus, the *Opn4^Cre/+^*;*Z/AP *reveals that the ipRGCs that innervate classical visual targets such as the dLGN follow a developmental paradigm similar to other RGCs, while the *Opn4^tauLacZ/+ ^*labeled ipRGCs appear to innervate their specific targets later.

To explore the functional output of the differing ipRGC projections to the OPN, we measured the PLR of postnatal mice. We first detected a rudimentary PLR at P7, coincident with the appearance of the *Opn4^tauLacZ/+^*-labeled axons in the OPN shell. Although we cannot rule out the role that other relay centers play in determining the onset of PLR, it is striking that the onset of PLR coincides with the appearance of *Opn4^tauLacZ/+^*-labeled axons in the OPN, a crucial connection for the PLR.

This early PLR must be driven by melanopsin since it accounts for all retinal photoreception through at least P10 [17,33,34] and synaptic connections between retinal layers are not functional until eye opening at P12 to P14 [35]. While it remains unclear why PLR begins several days prior to eye opening, it agrees with mounting evidence that ipRGCs comprise the first functioning photoreceptive system during development [17,34].

## Conclusions

Together, these data indicate diversity in the developmental trajectory of ipRGCs and form a foundation to explore the molecular mechanisms that govern the specification and wiring of this distinct visual subsystem.

## Materials and methods

### Animals

All procedures were performed on mice bred on a mixed C57BL/6x129 background in accordance with the IACUC protocols of Johns Hopkins University and Virginia Commonwealth University Medical Center. All mice were housed in a 12:12 hour light:dark cycle.

### X-gal staining

Tissue from *Opn4^tauLacZ/+ ^*mice was prepared as previously described [6], stained for 2 days in X-gal and counterstained with Vector Nuclear Fast Red.

### Alkaline phosphatase labeling

We used previously generated mice that express Cre recombinase from the endogenous melanopsin locus [7]. The experimental animals used in this study were obtained by mating *Opn4^Cre/Cre ^*mice to Z/AP reporter mice [13].

### Eye injections

To visualize retinofugal projections originating from each eye, intravitreal injections of the fluorescently conjugated anterograde tracer CTB were performed as described in [36]. Animals were given an 18- to 36-hour survival period.

### Melanopsin immunolabeling

Tissue was cryosectioned at 16 μm in the coronal plane and stained with rabbit α-melanopsin (1:2,000; a gift from I Provencio) and incubated on slides for 4 days at 4°C. Slides were incubated with donkey-anti-rabbit Alexa 488 (1:500; Molecular Probes; Carlsbad, CA USA). Slides were mounted in AntiFade (Molecular Probes) with DAPI.

### Birthdating

Females pregnant with *Opn4^tauLacZ/+ ^*pups were injected with EdU (Invitrogen; Carlsbad, CA USA) every 3 hours over a 24-hour period [12]. Tissue was collected at P0, sectioned at 18 μm in the coronal plane, and stained with chicken anti-β-galactosidase (1:600; Millipore; Billerica, MA USA) or mouse anti-Brn3a (1:25; Millipore) incubated overnight at 4°C followed by donkey anti-chicken Alexa 546 (1:500; Molecular Probes) or goat anti-mouse Alexa 546 (1:500; Molecular Probes). Sections were imaged at 40× with a Zeiss LSM 510 META confocal microcsope. The proportion of ipRGCs or Brn3a-positive cells born on a specific day was determined for samples of more than 100 cells per retina.

### Pupillary light response

PLR was evoked using a 473-nm light-emitting diode at 1.35 × 10^16 ^photons/cm^2^·s, [37]. To view the pupil before natural eye opening (P12 to P14), it was necessary to separate the eyelids along the line of fusion and gently hold them open with curved forceps. All animals were dark-adapted for at least 1 hour before measurements, which were restricted to the middle of the light portion of the day. One eye of each mouse was monitored under infrared light with a Sony DCR-HC96 video camera. The percentage of pupil constriction was calculated by comparing the pupil area at the end of each treatment to the pupil area in the dark at the beginning of the recording. To measure any baseline constriction due to handling or other non-light stimuli, percent constriction was measured after holding the mouse in the dark for 20 seconds. To measure the percent constriction due to light, the pupil area was measured after 30 seconds of light exposure in the opposite eye. To measure maximal pupil constriction, 1 to 2 μl of 100 mM carbachol was applied to one eye 5 minutes before measurement (Figure 4D). Individual video frames were captured from the beginning and end of the 20 seconds recorded in the dark, at the end of the 30 seconds of light, and 5 minutes after the application of carbachol. Topical application of 0.5% proparacaine was used to determine if the baseline constriction was due to sensation of the forceps used to hold the eye open.

## Abbreviations

CTB: cholera toxin B subunit; dLGN: dorsal lateral geniculate nucleus; E: embryonic day; EdU: 5-ethynyl-2'-deoxyuridine; ipRGC: intrinsically photosensitive retinal ganglion cell; LGN: lateral geniculate nucleus; OPN: olivary pretectal nucleus; P: postnatal day; PLR: pupillary light response; RGC: retinal ganglion cell; SCN: suprachiasmatic nucleus.

## Competing interests

The authors declare that they have no competing interests.

## Authors' contributions

DSM and CJS wrote the manuscript. CJS provided data for Figure 1A-D. DSM and JLE provided data for Figure 1E, 2B, and 4B. DSM provided data for Figure 1F,G, 2C, and 4C-F. TCB provided data for Figure 2E,F. DM and WG provided data for Figure 2A,D, and 4A. TCB, WG, and SH edited the manuscript.

## Supplementary Material

Additional file 1 **RGC and ipRGC birthdating**. **(A,B) **Series of representative images for birthdating of β-galactosidase-positive ipRGCs (A) and Brn3a-positive RGCs (B) at P0. Yellow arrowheads denote EdU-positive ipRGCs or Brn3a-positive RGCs, and white arrows denote EdU-negative cells. **(C,D) **Raw cell counts and proportions of EdU-positive ipRGCs (C) and Brn3a-positive RGCs (D). Scale bars: 50 μm. Click here for file

## References

[B1] Hatori M Le H Vollmers C Keding SR Tanaka N Schmedt C Jegla T Panda S Inducible ablation of melanopsin-expressing retinal ganglion cells reveals their central role in non-image forming visual responses PLoS ONE 2008 3 e2451 10.1371/journal.pone.0002451 18545654 PMC2396502

[B2] Goz D Studholme K Lappi DA Rollag MD Provencio I Morin LP Targeted destruction of photosensitive retinal ganglion cells with a saporin conjugate alters the effects of light on mouse circadian rhythms PLoS ONE 2008 3 e3153 10.1371/journal.pone.0003153 18773079 PMC2519834

[B3] Altimus CM Guler AD Villa KL McNeill DS Legates TA Hattar S Rods-cones and melanopsin detect light and dark to modulate sleep independent of image formation Proc Natl Acad Sci USA 2008 105 19998 20003 10.1073/pnas.0808312105 19060203 PMC2596746

[B4] Guler AD Ecker JL Lall GS Haq S Altimus CM Liao HW Barnard AR Cahill H Badea TC Zhao H Hankins MW Berson DM Lucas RJ Yau KW Hattar S Melanopsin cells are the principal conduits for rod-cone input to non-image-forming vision Nature 2008 453 102 105 10.1038/nature06829 18432195 PMC2871301

[B5] Hattar S Liao HW Takao M Berson DM Yau KW Melanopsin-containing retinal ganglion cells: architecture, projections, and intrinsic photosensitivity Science 2002 295 1065 1070 10.1126/science.1069609 11834834 PMC2885915

[B6] Hattar S Kumar M Park A Tong P Tung J Yau KW Berson DM Central projections of melanopsin-expressing retinal ganglion cells in the mouse J Comp Neurol 2006 497 326 349 10.1002/cne.20970 16736474 PMC2885916

[B7] Ecker JL Dumitrescu ON Wong KY Alam NM Chen SK LeGates T Renna JM Prusky GT Berson DM Hattar S Melanopsin-expressing retinal ganglion-cell photoreceptors: cellular diversity and role in pattern vision Neuron 2010 67 49 60 10.1016/j.neuron.2010.05.023 20624591 PMC2904318

[B8] Quina LA Pak W Lanier J Banwait P Gratwick K Liu Y Velasquez T O'Leary DD Goulding M Turner EE Brn3a-expressing retinal ganglion cells project specifically to thalamocortical and collicular visual pathways J Neurosci 2005 25 11595 11604 10.1523/JNEUROSCI.2837-05.2005 16354917 PMC6726022

[B9] Baver SB Pickard GE Sollars PJ Pickard GE Two types of melanopsin retinal ganglion cell differentially innervate the hypothalamic suprachiasmatic nucleus and the olivary pretectal nucleus Eur J Neurosci 2008 27 1763 1770 10.1111/j.1460-9568.2008.06149.x 18371076

[B10] Young RW Cell death during differentiation of the retina in the mouse J Comp Neurol 1984 229 362 373 10.1002/cne.902290307 6501608

[B11] Farah MH Easter SS Jr Cell birth and death in the mouse retinal ganglion cell layer J Comp Neurol 2005 489 120 134 10.1002/cne.20615 15977166

[B12] Rachel RA Dolen G Hayes NL Lu A Erskine L Nowakowski RS Mason CA Spatiotemporal features of early neuronogenesis differ in wild-type and albino mouse retina J Neurosci 2002 22 4249 4263 12040030 10.1523/JNEUROSCI.22-11-04249.2002 PMC4127325

[B13] Lobe CG Koop KE Kreppner W Lomeli H Gertsenstein M Nagy A Z/AP, a double reporter for cre-mediated recombination Dev Biol 1999 208 281 292 10.1006/dbio.1999.9209 10191045

[B14] Fahrenkrug J Nielsen HS Hannibal J Expression of melanopsin during development of the rat retina Neuroreport 2004 15 781 784 10.1097/00001756-200404090-00008 15073514

[B15] Mason CA Sparrow N Lincoln DW Structural features of the retinohypothalamic projection in the rat during normal development Brain Res 1977 132 141 148 10.1016/0006-8993(77)90711-9 890472

[B16] Speh JC Moore RY Retinohypothalamic tract development in the hamster and rat Brain Res Dev Brain Res 1993 76 171 181 10.1016/0165-3806(93)90205-O 8149583

[B17] Sekaran S Lupi D Jones SL Sheely CJ Hattar S Yau KW Lucas RJ Foster RG Hankins MW Melanopsin-dependent photoreception provides earliest light detection in the mammalian retina Curr Biol 2005 15 1099 1107 10.1016/j.cub.2005.05.053 15964274 PMC4316668

[B18] Badea TC Cahill H Ecker J Hattar S Nathans J Distinct roles of transcription factors brn3a and brn3b in controlling the development, morphology, and function of retinal ganglion cells Neuron 2009 61 852 864 10.1016/j.neuron.2009.01.020 19323995 PMC2679215

[B19] Young MJ Lund RD The anatomical substrates subserving the pupillary light reflex in rats: origin of the consensual pupillary response Neuroscience 1994 62 481 496 10.1016/0306-4522(94)90381-6 7830893

[B20] Radel JD Das S Lund RD Development of light-activated pupilloconstriction in rats as mediated by normal and transplanted retinae Eur J Neurosci 1992 4 603 615 10.1111/j.1460-9568.1992.tb00169.x 12106324

[B21] Drager UC Birth dates of retinal ganglion cells giving rise to the crossed and uncrossed optic projections in the mouse Proc R Soc Lond B Biol Sci 1985 224 57 77 10.1098/rspb.1985.0021 2581263

[B22] Brown NL Kanekar S Vetter ML Tucker PK Gemza DL Glaser T Math5 encodes a murine basic helix-loop-helix transcription factor expressed during early stages of retinal neurogenesis Development 1998 125 4821 4833 9806930 10.1242/dev.125.23.4821

[B23] Wee R Castrucci AM Provencio I Gan L Van Gelder RN Loss of photic entrainment and altered free-running circadian rhythms in math5-/- mice J Neurosci 2002 22 10427 10433 12451142 10.1523/JNEUROSCI.22-23-10427.2002 PMC6758748

[B24] Godement P Salaun J Imbert M Prenatal and postnatal development of retinogeniculate and retinocollicular projections in the mouse J Comp Neurol 1984 230 552 575 10.1002/cne.902300406 6520251

[B25] Edwards MA Schneider GE Caviness VS Jr Development of the crossed retinocollicular projection in the mouse J Comp Neurol 1986 248 410 421 10.1002/cne.902480309 3722464

[B26] Gooley JJ Lu J Chou TC Scammell TE Saper CB Melanopsin in cells of origin of the retinohypothalamic tract Nat Neurosci 2001 4 1165 10.1038/nn768 11713469

[B27] Shimomura H Moriya T Sudo M Wakamatsu H Akiyama M Miyake Y Shibata S Differential daily expression of Per1 and Per2 mRNA in the suprachiasmatic nucleus of fetal and early postnatal mice Eur J Neurosci 2001 13 687 693 10.1046/j.0953-816x.2000.01438.x 11207804

[B28] Shibata S Moore RY Development of neuronal activity in the rat suprachiasmatic nucleus Brain Res 1987 431 311 315 3040191 10.1016/0165-3806(87)90220-3

[B29] Pickard GE Bifurcating axons of retinal ganglion cells terminate in the hypothalamic suprachiasmatic nucleus and the intergeniculate leaflet of the thalamus Neurosci Lett 1985 55 211 217 10.1016/0304-3940(85)90022-9 4000547

[B30] Millhouse OE Optic chiasm collaterals afferent to the suprachiasmatic nucleus Brain Res 1977 137 351 355 10.1016/0006-8993(77)90345-6 589458

[B31] Prichard JR Stoffel RT Quimby DL Obermeyer WH Benca RM Behan M Fos immunoreactivity in rat subcortical visual shell in response to illuminance changes Neuroscience 2002 114 781 793 10.1016/S0306-4522(02)00293-2 12220578

[B32] Okoyama S Moriizumi T Onset of calbindin-D 28K and parvalbumin expression in the lateral geniculate complex and olivary pretectal nucleus during postnatal development of the rat Int J Dev Neurosci 2001 19 655 661 10.1016/S0736-5748(01)00047-8 11705670

[B33] Tu DC Zhang D Demas J Slutsky EB Provencio I Holy TE Van Gelder RN Physiologic diversity and development of intrinsically photosensitive retinal ganglion cells Neuron 2005 48 987 999 10.1016/j.neuron.2005.09.031 16364902

[B34] Johnson J Wu V Donovan M Majumdar S Renteria RC Porco T Van Gelder RN Copenhagen DR Melanopsin-dependent light avoidance in neonatal mice Proc Natl Acad Sci USA 2010 107 17374 17378 10.1073/pnas.1008533107 20855606 PMC2951438

[B35] Takada Y Fariss RN Tanikawa A Zeng Y Carper D Bush R Sieving PA A retinal neuronal developmental wave of retinoschisin expression begins in ganglion cells during layer formation Invest Ophthalmol Vis Sci 2004 45 3302 3312 10.1167/iovs.04-0156 15326155

[B36] Jaubert-Miazza L Green E Lo FS Bui K Mills J Guido W Structural and functional composition of the developing retinogeniculate pathway in the mouse Vis Neurosci 2005 22 661 676 10.1017/S0952523805225154 16332277

[B37] Lucas RJ Hattar S Takao M Berson DM Foster RG Yau KW Diminished pupillary light reflex at high irradiances in melanopsin-knockout mice Science 2003 299 245 247 10.1126/science.1077293 12522249

