# Supplementary figures and images for: Development of melanopsin-based irradiance detecting circuitry
Source: Neural Dev. 2011 Mar 18;6:8. doi: 10.1186/1749-8104-6-8 (PMC3070623; doi:10.1186/1749-8104-6-8)

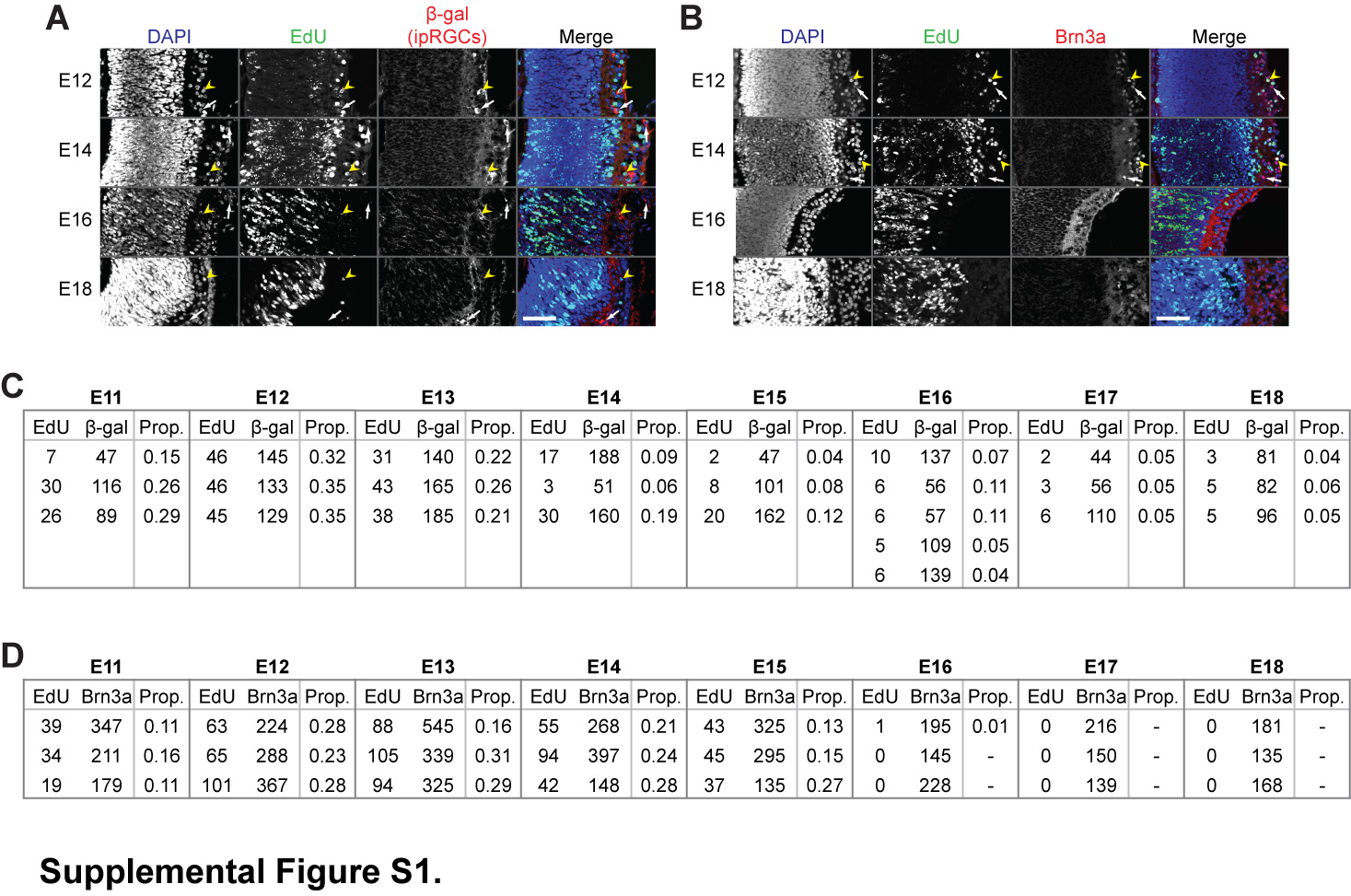

Supplement: Additional file 1 — RGC and ipRGC birthdating. (A,B) Series of representative images for birthdating of β-galactosidase-positive ipRGCs (A) and Brn3a-positive RGCs (B) at P0. Yellow arrowheads denote EdU-positive ipRGCs or Brn3a-positive RGCs, and white arrows denote EdU-negative cells. (C,D) Raw cell counts and proportions of EdU-positive ipRGCs (C) and Brn3a-positive RGCs (D). Scale bars: 50 μm. [file 1749-8104-6-8-S1.JPEG]
